# The Role of Transient Receptor Potential (TRP) Channels in the Transduction of Dental Pain

**DOI:** 10.3390/ijms20030526

**Published:** 2019-01-27

**Authors:** Mohammad Zakir Hossain, Marina Mohd Bakri, Farhana Yahya, Hiroshi Ando, Shumpei Unno, Junichi Kitagawa

**Affiliations:** 1 Department of Oral Physiology, School of Dentistry, Matsumoto Dental University, 1780 Gobara Hirooka, Shiojiri, Nagano 399-0781, Japan; shumpei.unno@mdu.ac.jp (S.U.); junichi.kitagawa@mdu.ac.jp (J.K.); 2 Department of Oral and Craniofacial Sciences, Faculty of Dentistry, University of Malaya, Kuala Lumpur 50603, Malaysia; marinab@um.edu.my (M.M.B.); farhanayahya@um.edu.my (F.Y.); 3 Department of Biology, School of Dentistry, Matsumoto Dental University, 1780 Gobara, Hirooka, Shiojiri, Nagano 399-0781, Japan; hiroshi.ando@mdu.ac.jp

**Keywords:** dental pain, dentine hypersensitivity, pulpitis, TRP channels, dental primary afferent neurons, odontoblasts, transduction mechanism

## Abstract

Dental pain is a common health problem that negatively impacts the activities of daily living. Dentine hypersensitivity and pulpitis-associated pain are among the most common types of dental pain. Patients with these conditions feel pain upon exposure of the affected tooth to various external stimuli. However, the molecular mechanisms underlying dental pain, especially the transduction of external stimuli to electrical signals in the nerve, remain unclear. Numerous ion channels and receptors localized in the dental primary afferent neurons (DPAs) and odontoblasts have been implicated in the transduction of dental pain, and functional expression of various polymodal transient receptor potential (TRP) channels has been detected in DPAs and odontoblasts. External stimuli-induced dentinal tubular fluid movement can activate TRP channels on DPAs and odontoblasts. The odontoblasts can in turn activate the DPAs by paracrine signaling through ATP and glutamate release. In pulpitis, inflammatory mediators may sensitize the DPAs. They could also induce post-translational modifications of TRP channels, increase trafficking of these channels to nerve terminals, and increase the sensitivity of these channels to stimuli. Additionally, in caries-induced pulpitis, bacterial products can directly activate TRP channels on DPAs. In this review, we provide an overview of the TRP channels expressed in the various tooth structures, and we discuss their involvement in the development of dental pain.

## 1. Introduction

Dental pain or odontogenic pain is the pain that initiates from the teeth or their supporting structures. The most common cause of dental pain is dental caries or tooth decay, the worldwide prevalence of which is very high. It was reported that in 2010, dental caries in permanent teeth remained the most prevalent global health problem, affecting 2.4 billion people, and dental caries in deciduous teeth constituted the tenth most prevalent health condition, affecting 621 million children worldwide [1]. Untreated dental caries lead to pulpitis (inflammation of the dental pulp) [2,3,4,5,6]. Typically, pulpitis is caused by invasion of the commensal oral microorganisms into the pulp due to caries [2,3]. Irritation of the dental pulp by mechanical, chemical, thermal or electrical stimuli may also cause pulpal inflammation [2,3,4,5,6]. Other causes of pulpitis include trauma, cracks on the tooth and periodontal infections [4,6]. Symptomatic pulpitis can be an extremely painful condition and is one of the main reasons that patients seek dental treatment [4,6,7]. It is often associated with intense lingering pain to thermal stimuli. The pain can be spontaneous, diffuse or referred [4,6,7].

Dentine hypersensitivity is another common odontogenic pain condition, the prevalence of which varies widely, ranging from 3–98% [8,9,10,11,12,13,14]. It is characterized by short, sharp pain arising from exposed dentine in response to stimuli—typically, thermal, evaporative, tactile, osmotic or chemical—and which cannot be ascribed to any other form of dental defect or disease [13,14,15]. The dentine can be exposed by chemical erosion, mechanical abrasion/attrition of enamel, and by loss of cementum following gingival recession [6,13,14,15]. The modern lifestyle increases the consumption of acidic foods and drinks that can result in significant tooth wear and exposure of dentine on any aspect of the tooth surface [13,14,15,16,17]. Dentine hypersensitivity is a special condition where dental pain arises in response to non-noxious stimuli on the exposed dentine that normally does not elicit pain in healthy teeth [6,13,14,15]. Even light tactile stimuli (weak air puff or water spray directed to the exposed dentine), which may only produce light touch sensation on the oral mucosa or skin, provokes abrupt intense pain [6,13,14,15]. There are three widely-held theories on the pathogenesis of this type of pain: (1) Dentinal fluid hydrodynamic theory, in which it has been proposed that external stimuli cause movement of the dentinal fluid that ultimately excites the nerve fibers in the pulp and initiates pain; (2) Neural theory, in which it has been suggested that the nerve fibers that project into the dentinal tubules directly respond to external stimuli; (3) Odontoblast transducer theory, in which odontoblasts themselves have been suggested as pain transducers [13,14,15,18,19,20]. Among these, the dentinal fluid hydrodynamic theory is the most widely accepted, although not without controversy [16,18,19,20,21,22,23,24,25,26,27]. In one study, water application onto human dentine did not evoke pain; however, it caused dentinal tubular fluid movement in vitro [24]. Another study demonstrated a lack of correlation between dentinal fluid flow and pain in patients after cold stimulation of the exposed dentine, suggesting that cold-sensitive receptors might also be involved in pain transduction [26]. Recently, based on their findings, Shibukawa et al. proposed the “odontoblast receptor hydrodynamic theory”, in which they posit that the movement of the dentinal fluid mechanically stimulates mechanoreceptors in both odontoblasts and the nerve fibers in the pulp [27]. Odontoblasts, movement of dentinal tubular fluid and nerves in the dental pulp may all be involved in dentine hypersensitivity; however, the underlying mechanisms are not yet fully understood [15].

In addition to painful pulpitis and dentine hypersensitivity, pain may also occur when intense thermal stimuli are applied on the surface of a normal intact tooth [28,29,30,31]. In the clinic, thermal pulp testing (applying heat or cold onto the tooth surface) is routinely used to test the vitality of the dental pulp of a tooth [28,29]. Thermal pulp testing induces a localized sharp pain in the tooth being tested if the tooth is vital [28,29,30,31]. Drinking/eating of very cold or hot drink/food may also induce dental pain [28,30,31].

Although dental pain is a common health problem, its molecular and cellular pathophysiology, particularly, how the external stimuli (e.g., physical, chemical or thermal) are transduced into electrical signals in the nerve that are ultimately perceived as pain, remain unclear. Various ion channels (e.g., voltage-sensitive Na^+^, mechanosensitive K^+^, L-type voltage-dependent Ca^2+^ channels) have been reported to be expressed in the dental pulpal afferents and in the odontoblasts, and may play an important role in the transduction process [32,33,34,35]. In recent decades, TRP channels have also been detected in odontoblasts and dental primary afferent neurons (DPAs), and have been implicated in the transduction of external stimuli into pain signals in the tooth [15,27,35,36]. This review focuses on the involvement of TRP channels in the transduction of dental pain.

## 2. Dental Innervation

**Figure 1:**
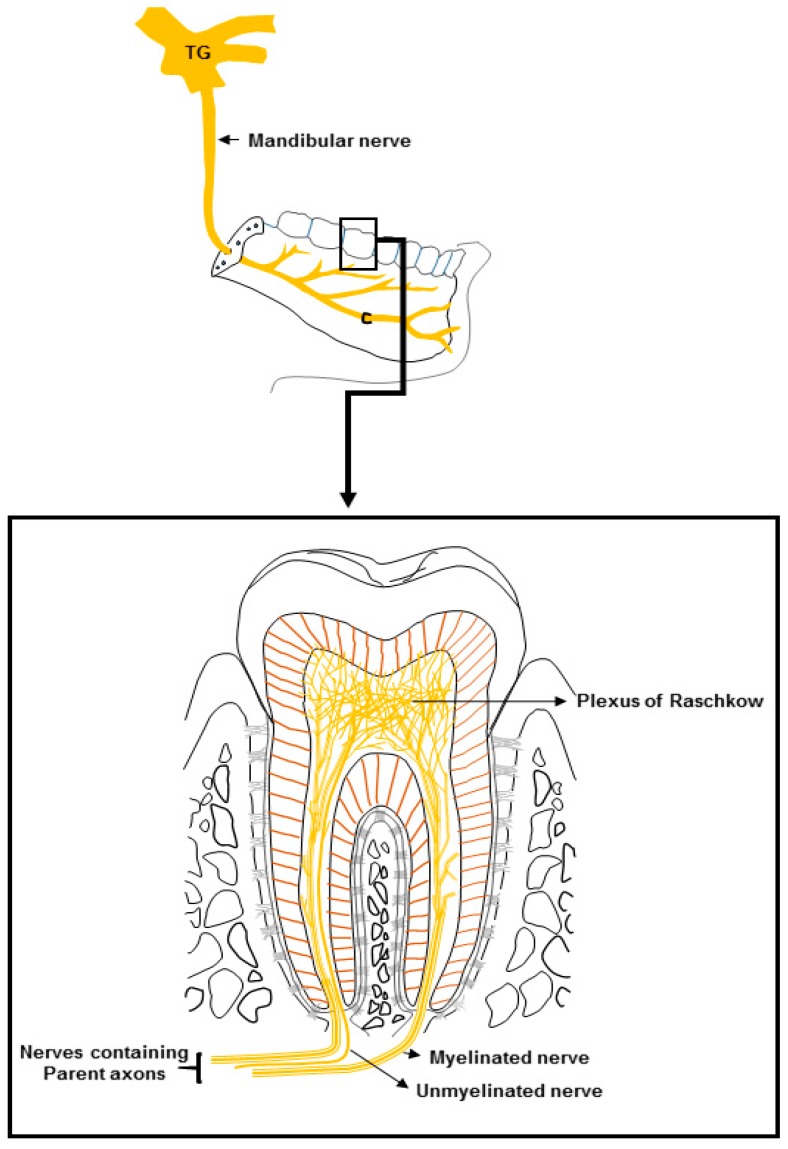
Innervation of a tooth. The cell bodies of the dental primary afferent neurons are located in the trigeminal ganglion (TG). The axons of the afferent neurons project to the dental pulp through the two major branches of the trigeminal nerve, namely, the mandibular (shown in the figure) and maxillary nerves. A large number of the parent axons of the afferent neurons before entering into the dental pulp are myelinated. After entering the dental pulp, they extend branches and gradually lose their myelin sheath. In the crown area, the axons branch extensively to form the plexus of Raschkow. Many axons terminate very close to the odontoblasts and sub-odontoblastic cells, and some enter the dentinal tubules for a short distance into the inner part of the dentine.

Teeth are highly vascularized and richly innervated structures [6,37,38]. The nerves of a tooth are mainly confined to the dental pulp [37,38]. The sensory stimuli-detecting nerve networks in the dental pulp differ in many ways from those in the skin or oral mucosa [34,39]. The various types of stimuli (e.g., mechanical, chemical or thermal) to the dental pulp or exposed dentine generally elicit only pain sensation, while these stimuli applied to the skin or oral mucosa produce distinct types of sensation [20,34,35,39,40]. The majority of the axons of the nerves that innervate a tooth enter the dental pulp through the apex [37,38,41,42]. Electron microscopic analysis of the nerve fibers within the dental pulp show that around 70–90% of the axons are unmyelinated (C-fibers) [40,41]. The remaining axons are mostly thinly myelinated (Aδ-fibers), and a very small amount are thickly myelinated (Aβ-fibers) [37,38,40,41]. However, animal studies in which retrograde labeling techniques were used to evaluate the size and histochemical composition of pulpal sensory neurons within the trigeminal ganglion (TG) suggest that the parent axons of the dental pulpal nerves are largely myelinated (Figure 1) [42,43,44].

It has been observed that the ratio of myelinated axons to unmyelinated axons is reduced in nerves closer to teeth, compared with more distant sites, indicating progressive loss of the myelin sheath as axons course toward the tooth [44,45]. Using electron microscopic analysis, a study reported that the percentage of unmyelinated axons was higher in the crown area compared with the root area in rat molars [46]. In that study, the unmyelinated axons of the dental pulp showed immunoreactivity to a marker of myelinated nerves, neurofilament (NF)-200, and the percentage of NF-200-positive unmyelinated axons was greater in the crown area than in the root area [46]. The conduction velocity in the nerve fibers outside the dental pulp was higher than in the nerve fibers located inside the pulp [47,48]. A study on human teeth also found that unmyelinated nerve fibers within the dental pulp show immunoreactivity to markers for myelinated nerves (neurofilament-200, neurofilament-52) [49]. Furthermore, studies have reported that the majority of parent axons before entering the dental pulp are thinly myelinated (Aδ), with a higher conduction velocity compared with nerve fibers inside the dental pulp [44,47,48,49]. These findings suggest that many parent axons in the dental pulp afferents are myelinated. Consistently, studies have reported that the cell bodies of the majority of the afferent fibers from the dental pulp that are located in the TG are medium-sized (Aδ-neurons), while a minority are large (Aβ-neurons) or small (C-neurons) [42,43]. After entering the dental pulp through the apex of the tooth, the parent axons of the afferent nerves extend branches and gradually lose their myelin sheath [43,46,49,50] (Figure 1). In the radicular pulp, the axons give off only a few branches, but in the coronal pulp, the axons branch extensively to form the plexus of Raschkow (Figure 1) [6,37,38,41]. The axons lose their myelin sheath mostly in the coronal pulp and emerge as free nerve endings (brush or fan-shaped) [49,50,51]. Many axons terminate very close to the odontoblasts, as well as sub- and peri-odontoblastic cells, and some enter the dentinal tubules and continue along odontoblast processes for a short distance, no further than 0.2 mm into the inner part of the dentine [51]. Some axons make endings in both the pulp and the dentinal tubule [51]. It has been reported that around 30–70% of odontoblast processes of a tooth are in close association with nerve endings in the inner part of the dentine [50]. The dental pulp also contains sparsely distributed sympathetic postganglionic efferent nerve fibers (unmyelinated), which mostly innervate the blood vessels, while parasympathetic nerve fibers have not been observed [40,41,52,53].

Cytochemically, the DPAs are distinct from the afferent neurons of the skin [34,39,43]. Generally, in the skin, the afferent neurons can be clearly divided into two major groups—peptidergic and non-peptidergic [54,55]. The peptidergic neurons express a variety of neuropeptides and signaling proteins, including calcitonin gene-related peptide (CGRP), substance P, nerve growth factor (NGF) receptor and tyrosine kinase A (TrkA) receptor, and these neurons are responsive to NGF. In comparison, the non-peptidergic neurons express isolectin B4 (IB4), glial cell line-derived neurotrophic factor (GDNF) family receptor alpha-1 (GFRα1) and receptors for other GDNF family members, and these neurons are responsive to GDNF [32,55,56,57]. The non-peptidergic neurons also express purinergic receptors (P2X3, a receptor for ATP) [58,59,60]. The pulpal afferents cannot be categorized into these two broad groups. They display the cytochemical features of both peptidergic and non-peptidergic neurons, which is rare for skin somatosensory afferents [61,62]. Although most pulpal afferents do not express IB4, they express other markers of non-peptidergic neurons such as GFRα1 and P2X3 [61,62], and a large group of pulpal afferents also express markers for peptidergic neurons such as CGRP, NGF receptor, substance P and TrkA receptors [63,64,65]. Another unique feature of dental pulp afferents is that they express markers for both mechanoreceptors and nociceptors [39,43,49,61,62]. It has been observed that pulpal nerve fibers express various cytochemical markers of mechanosensitive nerve fibers, such as neurofilament (NF) markers (e.g., NF-200, NF-52), parvalbumin, calbindin, epithelial sodium channels (ENaCs), acid-sensing ion channel 3 (ASIC3) and mechano-gated potassium channels [32,34,39,43,61,62,66,67]. They also express various cytochemical markers of nociceptive nerve fibers in the skin, such as CGRP, GFRα1, TrkA and substance P [39,43,61,68,69].

## 3. TRP Channels and Their Presence in Dental Tissues

TRPs are integral pore-forming membrane proteins that function primarily as non-selective ion channels [70,71,72,73]. They have a putative six-transmembrane-spanning protein domain with a pore region localized between transmembrane segments 5 and 6 [70,71,72,73]. They were discovered in the fruit fly (Drosophila) in studies of their phototransduction (light detection) mechanism [74,75,76]. Later, they were found in vertebrates, and to date, seven TRP subfamilies have been identified: TRPA (ankyrin), TRPC (canonical), TRPM (melastatin), TRPML (mucolipin), TRPP (polycystin), TRPN (Drosophila no mechanoreceptor potential C or NOMPC) and TRPV (vanilloid) [70,71,72,73,77]. Recently, the TRPY subfamily was reported in yeast [78]. TRPN and TRPY are absent in mammals [70,71,72,73,77]. The TRPV subfamily has six members (TRPV1–6), TRPA has one member (TRPA1), TRPC has seven members (TRPC1–7), TRPM has eight members (TRPM1–8), TRPML has three members (TRPML1–3), TRPP has three members (TRPP1–3), and TRPN has one member (TRPN1, found in fish) [70,71,72,73,77]. Among the 28 TRP channel genes that have been identified in mammals, 17 have been detected in the mouse TG at the mRNA level [79]. Many TRP channel genes have also been identified in the human TG [80].

To date, to our knowledge four members of the TRPV subfamily (TRPV1, TRPV2, TRPV3, TRPV4), four members of the TRPM subfamily (TRPM2, TRPM3, TRPM7, TRPM8), two members of the TRPC subfamily (TRPC1, TRPC6), and one member of the TRPA subfamily (TRPA1) have been detected in dental tissues (e.g., DPAs, odontoblasts).

### 3.1. TRPV

Among the six members of the TRPV subfamily, TRPV1–4 are weakly Ca^2+^-selective cation channels [70,71,72,73]. They can be activated by thermal stimulation, and are therefore referred to as thermo–TRPs [70,71,72,73,81]. In vitro studies show that TRPV1–4 can be activated by temperatures ranging from ~34 (TRPV4) to ~52 °C (TRPV2) [81]. The temperature range for channels activation is not strict, and a thermal threshold is not the optimal parameter to describe thermo-TRPs, because, the sensitivity for thermal activation of these channels is substantially modified by cellular and environmental factors [82,83,84]. For example, TRPV1 (normal activation temperature ~43 °C) can be activated at much lower temperatures when the membrane depolarized than when it hyperpolarized [82,83]. TRPV5–6 are highly Ca^2+^-selective and are not activated by heat. They play an important role in Ca^2+^ homeostasis [70,71,72,73].

TRPV1, which is also known as the capsaicin receptor, was the first member of the TRPV subfamily to be isolated [78]. It is activated by various exogenous and endogenous stimuli, such as capsaicin (found in hot chili peppers), acids (pH < 5.9), heat, inflammatory mediators (e.g., bradykinin, prostaglandins), NGF, anandamide (arachidonoyl ethanol amide), arachidonic acid metabolites (e.g., N-arachidonoyl-dopamine), lipoxygenase products (e.g., 12-hydroperoxyeicosatetraenoic acid), adenosine and ATP [85,86,87,88,89,90,91]. The threshold for activation of TRPV1 is dynamic [87,90,91]. For example, the threshold is decreased by inflammatory mediators, but after activation by capsaicin, there is a sustained refractory state (desensitization) [70,71,72,73,87,90,91]. Activation of TRPV1 has been shown to promote the release of neuropeptides such as substance P and CGRP [70,71,72,73,87,91]. TRPV1 is expressed predominantly in C-fibers and, to a lesser extent, in Aδ fibers [70,71,72,73,87,91]. In addition to sensory neurons, TRPV1 expression has been detected in various other tissues, including keratinocytes of the skin and oral mucosa, epithelium of the respiratory system, digestive tract, urinary bladder, cardiac muscle, vascular smooth muscle, and the endothelium of blood vessels [87,92]. TRPV1 has been reported to play an important role in thermal nociception [70,71,72,73,87,91]. The importance of TRPV1 in pain sensation has been demonstrated by studies in TRPV1 knockout mice [93]. It plays a role in many physiological functions, including satiety, olfaction, gastrointestinal motility, and energy homeostasis [72,87,92,93,94,95].

TRPV2 channels share 50% sequence identity with TRPV1 [70,73,81,96]. This channel has been reported to function as a thermoreceptor, mechanoreceptor and osmoreceptor [70,73,81,96,97]. TRPV2 is activated by noxious temperature (~52 °C) in vitro [97,98,99]. However, in vivo, TRPV2 knockout mice exhibit a thermal response similar to wild-type mice [100]. TRPV2 is expressed in the peripheral and central nervous systems [70,73,81,97,99,101,102,103,104]. In the spinal dorsal root ganglia (DRG) and in the trigeminal ganglia (TG), expression of TRPV2 was observed in the medium to large-diameter primary afferent neurons [97,99,102,103,104]. In culture, one-third of TRPV2-expressing DRG neurons are CGRP-positive, and activation of these neurons by a TRPV2 agonist results in CGRP release [102]. In the central nervous system, TRPV2 expression has been observed in a number of regions that are involved in osmoregulation and other autonomic functions [97,99,101].

TRPV3 can be activated by innocuous temperature (31–39 °C), various natural compounds (such as camphor and carvacrol), synthetic agents (such as 2-aminoethoxydiphenyl borate), and the endogenous ligand farnesyl pyrophosphate (FPP) [105,106,107,108]. TRPV3 expression in sensory neurons of the peripheral nervous system is not consistent across species, and it is minimally expressed in sensory neurons in the rodent; however, the channel is relatively abundantly expressed in skin keratinocytes [109,110,111,112]. TRPV3 is also expressed in the epithelial cells of the oral and nasal cavities, as well as the cornea, where it is reported to be involved in wound healing and thermo-sensation [111,112]. TRPV3 in keratinocytes is reported to be involved in nociception mediated by ATP [110]. Indeed, increased pain sensitivity is observed in transgenic mice overexpressing TRPV3 in skin keratinocytes [113].

TRPV4 was identified as an osmoreceptor [97,114,115,116]. Later, it was shown to be a polymodal receptor that can be activated by various stimuli, including innocuous warm temperature (27–35 °C) and mechanical stimuli (membrane stretch and shear stress) [97,117,118,119,120,121]. This channel can be activated by arachidonic acid metabolites, synthetic chemical agents such as 4α-phorbol 12,13-didecanoate, GSK1016790A, and the plant extract bisandrographolide A [70,72,73,122]. TRPV4 has also been implicated in nociception, itch, inflammatory pain, neuropathic pain and visceral pain [70,72,73,123,124,125,126,127]. Expression of TRPV4 is observed in DRG neurons [123,126,127] and TG [121,128,129,130] neurons. It is also expressed in satellite glial cells (cells surrounding the neurons) in the DRG that regulate neuronal excitability in pain and inflammatory conditions [131]. Inflammatory mediators (e.g., prostaglandins and proteases) may sensitize TRPV4, resulting in hyperalgesia [123,132]. Furthermore, escape latency in the hot plate test is increased following tissue injury and inflammation in TRPV4 knockout mice, suggesting involvement of TRPV4 in thermal hyperalgesia [124].

#### 3.1.1. TRPV in DPAs

TRPV channels have been detected in the cell bodies of DPAs located in the TG [133,134,135,136,137,138]. Among the six members of the TRPV subfamily, TRPV1 has been the most extensively studied [133,134,135,136,137,138,139,140,141]. In animals, the percentage of TRPV1-expressing dental pulp TG afferent neurons varies among the published papers from 8% to 85%, and TRPV1 expression is observed in small, medium and large neurons [133,134,135,136,137,138]. In 2001, Ichikawa & Sugimoto reported that approximately 8% of rat DPAs express TRPV1, and that 20% of these co-express the neuropeptide CGRP [133]. They also compared TRPV1-immunoreactive afferent neurons in the dental pulp and the facial skin [133]. The percentage of TRPV1-immunoreactive neurons was lower in the dental pulp (17%) than in the facial skin (26%) [133]. The same year, Chaudhary et al. reported TRPV1 mRNA expression in the cell bodies of DPAs in the TG, and 65% of these cells were excited by capsaicin. The capsaicin-evoked excitation was attenuated by a TRPV1 antagonist (capsazepine) [134]. Similar to Ichikawa & Sugimoto [133], Chung et al. reported that approximately 10% of the dental pulpal afferent neurons expressed TRPV1, and that the majority of these were small to medium neurons [135]. They also observed that TRPV1 expression was upregulated by application of lipopolysaccharides (a product of Gram-negative bacteria) into the dentine [135]. Stenholm et al. reported that 21–34% of dental pulpal afferent neurons were TRPV1-immunoreactive, slightly higher than among gingival primary afferent neurons (21–26%) [136]. Gibbs et al. found that approximately 17% of the afferent neurons in the TG from the dental pulp expressed TRPV1, lower than the percentage of TRPV1-immunoreactive afferent neurons from the periodontal ligament (26%) in the rat [104]. They also found that 70% of the TRPV1-immunoreactive neurons were myelinated, and that a majority (82%) of these were medium to large-diameter neurons [104]. Furthermore, 60% of the TRPV1-immunoreactive neurons co-expressed CGRP [104]. A high percentage of TRPV1-immunoreactive DPAs was observed by Kim et al. [137] and Park et al. [138]. Kim et al. showed that 85% of the retrograde-labeled rat DPAs were immunoreactive for TRPV1, and that 71% of them produced inward cationic currents in response to application of a TRPV1 agonist (capsaicin) [137]. Park et al. observed that 45% of the DPAs expressed TRPV1 [138]. They also showed that application of a TRPV1 agonist increased the intracellular Ca^2+^ concentrations and produced inward currents in these neurons. Temperature changes (>42 °C) also increased the intracellular Ca^2+^ concentrations and produced inward currents [138].

Expression of TRPV1 has been reported in the nerve fibers within the dental pulp in animal and human studies [137,139,140,141]. Kim et al. [137] found TRPV1-immunoreactive nerve fibers within the rat dental pulp; however, Gibbs et al. did not observe TRPV1-immunoreactive nerve fibers within the dental pulp of rat molars [104]. TRPV1 immunoreactivity has also been detected in the dental pulp nerve fibers of the human permanent teeth [139,140,141], and immunoreactivity is significantly increased in carious teeth compared with non-carious teeth [141]. Interestingly, TRPV1 expression tended to be increased in painful carious teeth compared with non-painful carious teeth, although the difference was not significant [141].

TRPV2 is expressed in the nerves within the dental pulp and in the cell bodies of the DPAs located in the TG [103,104,136]. In 2000, Ichikawa & Sugimoto reported TRPV2-immunoreactive nerve fibers within the rat dental pulp [103]. In the root area, TRPV2 immunoreactivity was observed in the nerve bundles, and in the crown area, the TRPV2-immunoreactive nerve fibers were ramified and extended to the base of the odontoblastic cell layer [103]. These investigators also observed that approximately 37% of the dental primary afferent neurons retrogradely traced in the TG expressed TRPV2, and that most of these were medium to large neurons [103]. In addition, 45% of these neurons co-expressed CGRP, and 41% co-expressed parvalbumin (a marker of proprioceptors) [103]. Furthermore, the percentage of TRPV2-immunoreactive afferent neurons (37%) was greater than the percentage of TRPV2-immunoreactive facial skin afferent neurons (9%) [103]. Stenholm et al. reported that 32–51% of DPAs expressed TRPV2, somewhat higher than the percentage of TRPV2-expressing gingival afferent neurons (41%) [136]. Gibbs et al. reported that a higher percentage of rat DPAs expressed TRPV2 compared with TRPV1; 50% were immunoreactive for TRPV2, while 17% were immunoreactive for TRPV1 [104]. They also observed that TRPV2 expression was higher among DPAs (50%) compared with primary afferent neurons from the periodontal ligament (41%) [104]. Approximately 83% of the TRPV2-immunoreactive neurons were myelinated, and the majority of these were medium to large neurons [104]. Around 33% of the TRPV2-immunoreactive neurons co-expressed CGRP [104].

TRPV3 and TRPV4 are expressed in the TG on afferent neurons from the facial skin and temporomandibular joint [105,129,130]. TRPV4 expression is also observed on the nerves of human dental pulp, and expression is upregulated by chronic inflammation of the pulp [142].

#### 3.1.2. TRPV in Odontoblasts

In 2005, Okumura et al. reported expression of TRPV1 on odontoblasts in neonatal rat teeth [143]. They also showed a capsaicin-induced inward current in the odontoblasts using the patch clamp technique that was inhibited by capsaizepine [143]. However, in acutely isolated adult rat odontoblasts, Yeon et al. did not observe expression of TRPV1 by immunohistochemistry, and intracellular Ca^2+^ concentration in the odontoblasts was not increased by application of heat (42 °C) or a TRPV1 agonist (capsaicin) [144]. They also did not detect TRPV1 or TRPV2 mRNA in the odontoblasts [144]. In contrast, Tsumura et al. reported expression of TRPV1 in adult rat odontoblasts, on the cell membrane and on their processes [145]. Application of an agonist (capsaicin/resiniferatoxin/low pH solution) increased the intracellular Ca^2+^ level, which was inhibited by application of antagonists [145]. They also reported that the TRPV1 channels in odontoblasts are functionally coupled with cannabinoid receptor 1 and Na^+^–Ca^2+^ exchangers, and that this coupling is mediated by cyclic adenosine monophosphate (cAMP) [145]. TRPV1 has also been detected on the odontoblasts of extracted healthy caries-free human premolar teeth [139]. mRNAs for TRPV1, TRPV2, TRPV3 and TRPV4 were detected in acutely isolated cultured odontoblasts by Son et al. [146]. They also showed that when odontoblasts were stimulated by heat above 32 °C, the intracellular Ca^2+^ concentration increased, suggesting functional involvement of TRPV1, TRPV2 and TRPV3 in the transduction of heat stimuli [146]. In addition, the intracellular Ca^2+^ concentration was increased when hypotonic solution was applied, suggesting involvement of TRPV4 channels [146]. Sato et al. reported expression of TRPV2 and TRPV4 proteins in rat odontoblasts [147]. They also showed that extracellular hypotonic solution-induced membrane stretching in cultured mouse odontoblast lineage cells produces inward currents and increases intracellular Ca^2+^ concentration, which can be inhibited by application of TRPV1, TRPV2 and TRPV4 channel antagonists, suggesting that these channels function as mechanoreceptors in odontoblasts [147]. TRPV1 and TRPV4 expression has been reported by immunohistochemistry, PCR and western blot analysis in human odontoblast-like cells derived from the dental pulp of a permanent tooth [148,149]. Application of agonists to these channels increases the intra-odontoblast Ca^2+^ concentration. Hypotonic solution-induced membrane stretch also increases the intra-odontoblastic Ca^2+^ concentration, which can be reduced by channel antagonists [148,149]. Brief (10 min) application of the pro-inflammatory cytokine tumor necrosis factor (TNF)-α enhances the response to chemical agonists and hypotonic solution-induced membrane stretch, suggesting that inflammation increases TRPV4 channel activation [148,149]. TRPV4 immunoreactivity has been detected in odontoblasts and their process in extracted healthy caries-free immature human third molar teeth [149]. Wen et al. reported expression of TRPV1, TRPV2 and TRPV3 in native human odontoblasts as well as cultured odontoblast-like cells from healthy human third molars by immunohistochemistry, quantitative real-time polymerase chain reaction (qRT-PCR), western blotting and immunoelectron microscopy [150]. Immunoelectron microscopy revealed expression of TRPV1, TRPV2 and TRPV3 in odontoblastic processes, mitochondria and endoplasmic reticulum [150]. Egbuniwe et al. reported expression of TRPV1 and TRPV4 mRNAs in odontoblast-like cells derived from human immortalized dental pulp cells. They observed that the application of a TRPV4 agonist increased the intra-odontoblastic Ca^2+^ concentration. They also observed that the agonist caused release of ATP from the odontoblasts, which was blocked by pretreatment with the antagonist [151]. In odontoblast-like cells obtained from cultured dental pulp cells from newborn rats, mechanical stimulation increases the intracellular Ca^2+^ concentration, and this effect can be attenuated by application of TRPV1, TRPV2 and TRPV4 antagonists, suggesting that these channels function as mechanosensors in these cells [27].

### 3.2. TRPM

TRPM8 has been detected in DPAs and pulpal fibroblasts. TRPM3 and TRPM7 are expressed in odontoblasts, while TRPM2 is expressed in pulpal fibroblasts. TRPM2 is activated by cellular stress and participates in various cellular functions, including cytokine production, cell motility and cell death [152,153]. It can be activated by cytosolic adenosine diphosphate ribose (ADPR), oxidative stress and moderate heat in various cell types [152,153,154,155]. TRPM2 expression has also been detected in afferent sensory neurons in the DRG and TG [79]. This channel is implicated in pathogenic pain [156].

TRPM3 is expressed in non-neural (e.g., epithelium of the kidney, pancreatic β cells) and neural tissues, including sensory neurons in the DRG and TG [157,158,159,160,161]. TRPM3 can be activated by extracellular hypo-osmolarity, noxious heat (>30 °C) and chemical compounds, such as pregnenolone sulfate (endogenous excitatory neurosteroid) and nifedipine (a drug used for hypertension) [157,160,161]. TRPM3 knockout mice exhibit significant attenuation of thermal hyperalgesia under inflammatory conditions [160].

Similar to other TRP channels, TRPM7 is expressed in a wide variety of tissues, including the brain, heart and hematopoietic tissues [162,163,164,165,166]. TRPM7 has been implicated in multiple cellular and physiological functions, including embryonic development, Mg^2+^ homeostasis, cell growth and viability, synaptic transmission, and neuronal degeneration [163,164,165,166].

TRPM8 is activated by innocuous cooling (~26−15 °C) as well as by noxious cooling (<15 °C) and by a number of cooling agents, such as menthol and icilin [167,168]. TRPM8 is expressed in small-diameter sensory neurons in the TG and DRG [169,170,171,172]. TRPM8 mRNA is more abundantly expressed in the sensory afferents of the TG (especially those that innervate the tongue) compared with the DRG [169,170,171,172]. While TRPM8 knockout mice do not lack sensation to noxious cold, they exhibit an attenuation of avoidance behavior to moderately cold temperatures [173,174]. These mice also display reduced cold hypersensitivity following nerve injury or complete Freund’s adjuvant (CFA)-induced inflammation [175].

#### 3.2.1. TRPM in DPAs

TRPM8 is expressed in rat DPAs in the TG [137,138,176]. By immunohistochemistry, Park et al. observed that 13% of rat DPAs expressed TRPM8 [138]. Application of menthol (a TRPM8 agonist) or exposure to temperatures less than 25 °C increase intracellular Ca^2+^ concentrations and evoke inward currents in the TRPM8-expressing neurons [138]. In one study, TRPM8 mRNA expression was detected in 58% of rat DPAs [137]. However, Michot et al. detected TRPM8 expression in only 5.7% of mouse DPAs, similar to that in afferent neurons innervating the facial skin and the buccal mucosa [176].

#### 3.2.2. TRPM in Odontoblasts

Expression of TRPM3, TRPM8 and TRPM7 in odontoblasts has been reported in numerous studies [146,148,177,178,179,180,181,182]. Son et al. detected mRNA expression of osmo-sensitive TRPM3 channels in acutely isolated cultured odontoblasts from neonatal mice [146]. They also suggested that TRPM3 in these cells are involved in the transduction of osmotic stimuli, based on their finding of increased intra-odontoblastic Ca^2+^ concentration following exposure to hypotonic solution [146]. However, these investigators did not observe functional expression of TRPM8 [146]. TRPM8 expression was also not observed by Yeon et al. in acutely isolated adult rat odontoblasts at the mRNA level [144]. They showed that application of cold stimuli (12 °C) or menthol (TRPM8 agonist) did not increase the intra-odontoblastic Ca^2+^ concentration [144]. In contrast, Tsumura et al. reported functional expression of TRPM8 in acutely isolated adult rat odontoblasts [177]. TRPM8 immunoreactivity was observed in the odontoblasts and their processes [177]. Chemical agonists (e.g., menthol, WS3, WS12) and temperature changes (22 ± 1 °C) also increased the intra-odontoblastic Ca^2+^ levels, and these changes could be reduced by an antagonist [177]. TRPM8 expression was also reported in odontoblast-like cells from the dental pulp of a human permanent tooth at both the mRNA (using PCR) and protein levels (using western blotting and immunohistochemistry) [148]. Chemical agonism also increased the intra-odontoblastic Ca^2+^ concentration in an antagonist-sensitive manner [148]. TRPM8 has also been detected in freshly isolated human odontoblasts at the mRNA and protein levels as well as in odontoblasts and their processes in extracted healthy caries-free human permanent teeth [178].

By single-cell RT-PCR and immunocytochemical analysis, TRPM7 was detected in acutely isolated rat odontoblasts [179,180]. Functionality of TRPM7 as a mechanoreceptor in acutely isolated rat odontoblasts was reported by Won et al. [180]. In their study, hypotonic solution-induced membrane stretch or chemical agonism for TRPM7 caused a transient increase in intra-odontoblastic Ca^2+^ concentration, which was blocked by a non-selective mechanosensitive channel blocker or a TRPM7 blocker [180]. TRPM7 in odontoblasts has also been reported to play an important role in dentine mineralization by regulating intracellular Mg^2+^ and alkaline phosphatase activity [181,182].

### 3.3. TRPA

The only member of the TRPA subfamily, TRPA1 is generally involved in pain, thermal and chemical sensation [70,73,91,97]. It is expressed in many tissues, including the brain, heart, small intestine, lung, bladder and joints [183,184,185]. It is highly expressed in small and medium neurons in the DRG and TG [91,183,186,187,188]. TRPA1 channels are co-localized with TRPV1, CGRP, substance P and bradykinin receptors [186,189,190,191]. TRPA1 is activated by noxious cold (<8 °C) and by exogenous chemical agents, such as allyl isothiocyanate (AITC, present in mustard oil and wasabi), cinnamaldehyde (found in cinnamon), allicin (found in garlic) and acrolein (present in diesel exhaust) [191,192,193].

#### 3.3.1. TRPA in DPAs

In rat DPAs (labeled using retrograde dye applied to the dental pulp), TRPA1 was observed in 11% of neurons [138]. Chemical stimulation by icilin (a TRPA1 and TRPM8 agonist) and cold stimulation (<17 °C) increase intracellular Ca^2+^ concentrations and produce inward currents in the TRPA1-expressing neurons [138]. Kim et al. [137] reported that 34% of rat DPAs expressed TRPA1 mRNA, and Hermanstyne et al. [34] observed TRPA1 mRNA expression in 64% of retrogradely-labeled DPAs in the rat. TRPA1 protein in the rat TG was increased following experimental exposure of the dental pulp, implicating the protein in hyperalgesia and allodynia following tooth injury [194]. In the mouse, TRPA1 was detected in 18.9% of DPAs, lower than in neurons innervating the buccal mucosa (43%) or the facial skin (24.6%) [176]. In the same study, noxious cold stimulation of the tooth increased the expression of c-Fos (a marker of neuronal excitation) in the brainstem trigeminal nucleus; however, this increase was not blocked by systemic administration of a TRPA1 antagonist [176].

TRPA1 is also expressed in a large number of axons that branch extensively in the peripheral pulp [195]. By electron microscopy, TRPA1 immunoreactivity was observed near the plasma membrane of unmyelinated axons. TRPA1 was also co-localized with a sodium channel, Nav1.8. Furthermore, TRPA1 expression on myelinated nerve fibers was upregulated in teeth with signs of pulpitis [195].

#### 3.3.2. TRPA in Odontoblasts

Tsumura et al. reported expression of TRPA1 on the cell membrane of acutely isolated adult rat odontoblasts and their processes [177]. The agonist AITC increased intracellular Ca^2+^ levels in an antagonist-sensitive manner [177]. Temperature changes (13 ± 1 °C) also similarly increased the intracellular Ca^2+^ level in a TRPA1 antagonist-sensitive manner. In addition, TRPA1 antagonists reduce the hypotonic solution-induced increase in intracellular Ca^2+^ level, suggesting that TRPA1 functions as a mechanoreceptor in odontoblasts [177]. TRPA1 was found to be expressed at both the mRNA and protein levels in human odontoblast-like cells derived from the dental pulp of a permanent tooth [149,196]. A TRPA1 agonist also increased the intra-odontoblast Ca^2+^ concentration in an antagonist-sensitive manner [149,196]. Gene silencing experiments showed that TRPA1 in odontoblasts functions as a receptor for noxious cold temperature. Hypotonic solution-induced membrane stretch and chemical agonism also increased the intra-odontoblastic Ca^2+^ concentration [149,196]. A brief (10 min) application of TNF-α enhanced these responses, suggesting that inflammatory conditions increase TRPA1 activity [149,196]. Long-term (24 h) application of TNF-α also enhanced TRPA1 activity and upregulated its mRNA and protein levels in odontoblast-like cells [149]. Furthermore, TRPA1 immunoreactivity in odontoblasts and their process is increased in carious teeth compared with caries-free human third molar teeth [149,196]. Egbuniwe et al. reported expression of TRPA1 mRNA in odontoblast-like cells from human immortalized dental pulp cells [151]. A TRPA1 agonist increased the intra-odontoblastic Ca^2+^ concentration and caused the release of ATP from these cells, and this effect was blocked by antagonist pretreatment [151]. TRPA1 is also detected on odontoblasts and their processes in extracted healthy caries-free human premolar teeth [195]. However, Tazawa et al. did not observe TRPA1 immunoreactivity on odontoblasts or their processes in extracted healthy caries-free human permanent teeth [178]. In acutely isolated adult rat odontoblasts, Yeon et al. also did not observe TRPA1 mRNA expression [144]. A study using odontoblast-like cells derived from cultured dental pulp cells of newborn rats found that mechanical stimulation increased the intra-odontoblastic Ca^2+^ concentration, which was attenuated by a TRPA1 antagonist, suggesting involvement of this channel in the transduction of mechanical stimuli [27].

### 3.4. TRPC

TRPC1 and TRPC6 have been reported to be expressed in odontoblasts [179,197,198]. TRPC1 plays an important role in store-operated Ca^2+^ entry (SOCE) in a variety of tissues and cell types [199,200]. TRPC1 is highly expressed in the hippocampus, amygdala, cerebellum, substantia nigra and inferior colliculus [201,202,203], and participates in important neuronal processes related to synaptic transmission and plasticity [203]. TRPC1 expression is observed on large myelinated sensory neurons in the DRG [204], and it and TRPC6 play critical roles in mechanosensation, hearing [205,206,207,208] and cell membrane stretch [206,207]. TRPC1 and TRPC6 are co-expressed with TRPV4 in the DRG, where they are implicated in mechanical hypersensitivity in inflammatory and neuropathic pain conditions [208].

#### TRPC in Odontoblasts

TRPC1 and TRPC6 mRNA expression has been reported in acutely isolated rat odontoblasts, where they have been suggested to play a role as mechanoreceptors [179]. TRPC1 and TRPC6 immunoreactivity is also detected on odontoblasts in human permanent teeth. TRPC6 protein expression increases with time during odontoblast differentiation from pulp tissue. Furthermore, differentiation is inhibited by downregulation of TRPC1 and TRPC6 expression, indicating involvement of these channels in the odontogenic differentiation of human dental pulp cells [197,198].

### 3.5. TRP Channels in Pulpal Fibroblasts and Blood Vessels

Expression of TRPV1 mRNA and protein has been reported in human dental pulp fibroblasts cultured from the pulp of third molar teeth [209], and application of the agonist capsaicin induces production of IL-6 (an inflammatory cytokines) in these cells, which is dose-dependently inhibited by the TRPV1 antagonist capsazepine, indicating involvement of this channel in fibroblast-mediated pulpal inflammation [209]. TRPV1 expression was also observed in pulpal fibroblasts of healthy caries-free human permanent teeth [139]. Karim et al. reported mRNA and protein expression of TRPA1 and TRPM8 in human dental pulp fibroblasts [210]. Application of the TRPM8 agonist menthol or cool temperature (22 ± 3 °C) in combination with the TRPA1 agonist cinnamaldehyde or noxious cold temperature (12 ± 2 °C) increased the intra-fibroblastic Ca^2+^ concentration, suggesting that TRPA1 and TRPM8 in pulp fibroblasts are involved in sensing environmental stimuli [210]. TRPM2 has also been detected in the fibroblasts of human dental pulp, and its expression is upregulated in teeth with signs of irreversible pulpitis [211].

## 4. Involvement of TRP Channels in the Transduction of Dental Pain

TRP channels in odontoblasts and DPAs are suggested to function as polymodal receptors involved in the transduction of various external stimuli. TRPV1, TRPV2, TRPV4, TRPM7 and TRPA1 in odontoblasts have been implicated as mechanoreceptors [27,146,147,148,149,151,180]. In an odontoblast/TG co-culture preparation, mechanical stimulation of the odontoblast-like cells increased intracellular Ca^2+^ concentration in the mechanically-stimulated odontoblasts as well as in neighboring neuron-like cells [27]. The increase in intracellular Ca^2+^ in these cells was almost completely abolished by application of a cocktail of TRPV1, TRPV2, TRPV4 and TRPA1 channel antagonists [27]. In addition, hypotonic solution-induced mechanical stretching of the cell membrane increases intracellular Ca^2+^ in cultured odontoblast-like cells [146,147,148,149,177,180], which is inhibited by antagonists of TRPV1, TRPV2, TRPV4 [146,148], TRPA1 [177] and TRPM7 [180], further suggesting that these channels function as mechanoreceptors and/or osmoreceptors.

**Figure 2:**
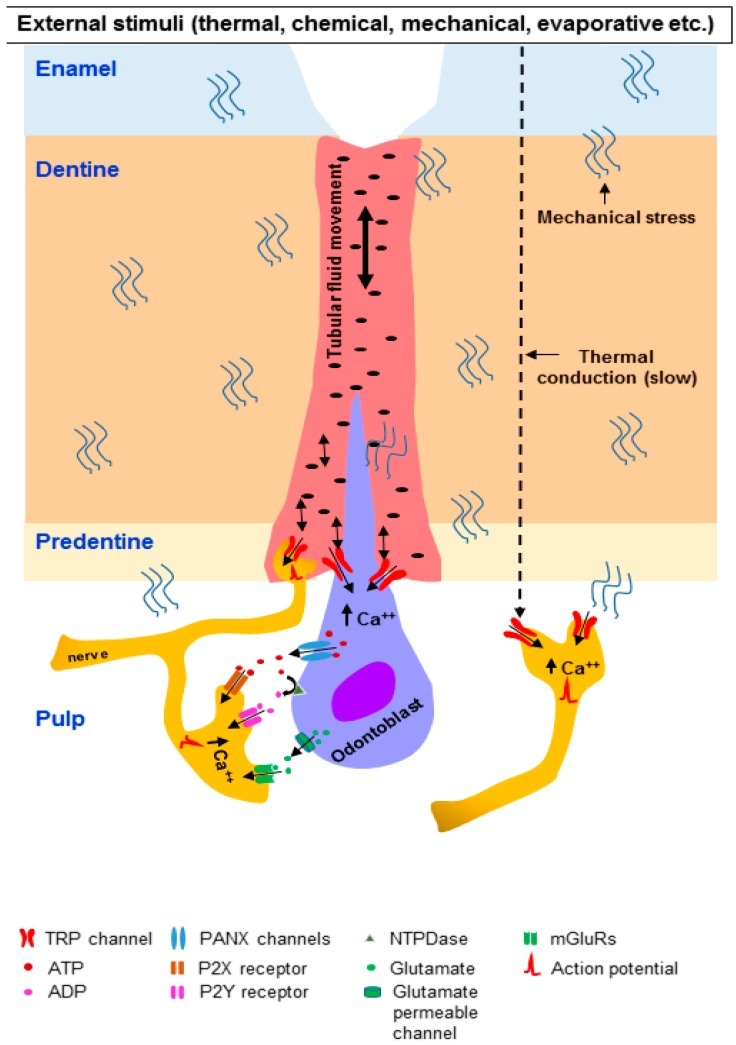
Mechanisms by which TRP channels may transduce dental pain when external stimuli are applied on the exposed dentine or on the surface of an intact tooth. External stimuli on the exposed dentine may create movement (indicated by double way arrows) in the dentinal tubular fluid which can activate the mechanosensitive TRP channels on odontoblasts and pulpal nerves. Intense thermal stimulation on the surface of an intact tooth may induce mechanical stresses within the tooth structures that ultimately excite the mechanosensitive TRP channels. In addition, temperature may conduct through the dental structures (relatively slow) to activate the thermosensitive TRP channels. Odontoblasts may communicate with the pulpal nerves through paracrine signaling mechanisms using ATP and glutamate. Ca^2+^ enters (indicated by single way arrows) odontoblasts through the activated TRP channels. ATP may be released (indicated by a single way arrow) from the odontoblasts through pannexin (PANX) channels and can activate P2X receptors expressed on the pulpal nerves. ATP can be converted (indicated by a curve arrow) by NTPDases to ADP, which can activate P2Y receptors expressed on the pulpal nerves. Furthermore, glutamate released (indicated by a single way arrow) from odontoblasts through glutamate-permeable channels can excite the pulpal nerves via metabotropic glutamate receptors (mGluRs).

Mechanosensitive TRP channels along with other mechanoreceptors expressed in odontoblasts and DPAs can be activated by application of thermal stimuli to an intact tooth. A study on human subjects showed that when a thermal stimulus was applied to a tooth surface, the subjects rapidly sensed pain (1.28 s for hot stimuli and 1.49 s for cold stimuli), even before a noticeable change in temperature at the wall of the dental pulp (3.68 s) [28,30]. Thermal stimulation on the tooth surface can induce fluid movement in the dentinal tubules because of thermal expansion or contraction of the fluid [18,212]. Interestingly, fluid movement was observed before a change in temperature in the dentine, where the dentinal tubules are located [21,22]. A temperature gradient between the enamel and dentine is observed when a thermal stimulus is applied on the surface of a tooth [21,22,23,25]. Tooth structures expand or contract because of this thermal gradient, which produces mechanical stresses in these structures [21,22,23,25,28]. Mechanical deformation of the dentine (including pulpal wall dentine) precedes the temperature changes in the dentine/dentine–enamel junction following thermal stimulation of the surface a tooth [22,25]. These observations suggest that intense thermal stimulation-induced mechanical deformation of the dentine may exert mechanical stresses on the odontoblasts as well as on the pulpal tissues. In turn, the mechanical stresses may directly activate the mechanosensitive TRP channels and other mechanoreceptors present in the odontoblasts and pulpal nerve fibers (Figure 2).

The sharp pain perceived soon after thermal stimulation of the tooth surface may be at least partly attributed to the mechanical stress-induced activation of mechanoreceptors on odontoblasts and DPAs. Temperature changes in the dentine caused by thermal stimulation of the surface of an intact tooth or a tooth with exposed dentine may also cause expansion/contraction and movement of dentinal tubular fluid, which can activate mechanosensitive TRP channels (along with other mechanoreceptors) on odontoblasts and nerve fibers within/near the dentinal tubules (Figure 2).

Intense thermal stimulation of the tooth surface may cause vasodilation and changes in pulpal blood flow [213,214,215]. A study using dogs found that pulpal blood flow increased slightly and gradually when the tooth surface temperature was raised from 35 to 40 °C, and that it increased sharply when the temperature was raised from 35 to 55 °C [213,214]. The increase of pulpal blood flow by thermal stimulation can increase the pressure within the pulpal tissue [213,214], which can excite mechanoreceptors, including TRP channels, in the pulpal nerves.

Patients with dentine hypersensitivity may feel pain caused by osmotic changes. Osmotic stimulation of the exposed dentine may induce movement of dentinal tubular fluid [18,19], which can excite mechanosensitive TRP channels on nearby odontoblasts and nerve fibers (Figure 2). It has been observed that dentine covered with a smear layer is much less responsive to hypertonic solutions than dentine devoid of a smear layer, suggesting that fluid movement is greater when the smear layer is absent [216].

Patients with dentine hypersensitivity may also feel pain after very light mechanical stimulation, such as air puffs or water spray, on the exposed dentine, which may induce very little movement of the dentinal tubular fluid. This suggests the presence of low-threshold mechanoreceptors on odontoblasts and DPAs [39]. Fried et al. proposed that many DPAs may contain low-threshold mechanoreceptors [39]. They termed these “algoneurons” [39]. The researchers suggested that these algoneurons may transduce the nociceptive signal in teeth [39]. Indeed, activation of these neurons causes activation of trigeminal brainstem neurons that deliver a pain message to higher brain centers, resulting in the sensation of pain [39]. Similar low-threshold mechanoreceptors in the skin or oral mucosa generally signal tactile sensation (e.g., light touch) [217]. Air puff on the exposed dentine may also dehydrate the dentine surface and cause outward flow of the dentinal tubular fluid, which can activate mechanosensitive receptors on odontoblasts and nerve fibers within/near the dentinal tubules [18,19]. Mechanosensitive TRP channels may function as mechanoreceptors [218] to transduce these types of stimuli.

When intense thermal stimuli are applied on the tooth surface, they can increase the temperature at the dentine–pulp border (albeit slowly), which may excite the thermosensitive TRP channels on odontoblasts and DPAs (Figure 2). Indeed, a slow increase in temperature to >43 °C on the tooth surface activates intra-pulpal C-fibers in the cat [219,220]. Another study showed that intra-dental A-δ and C-fibers respond to intense cooling of the tooth surface [31]. Thermosensitive TRP channels expressed on the A-δ and C-fibers in the dental pulp can be activated by high-temperature stimulation of the tooth surface. Heat and cool/cold stimuli increase intracellular Ca^2+^ concentrations and elicit inward currents in cultured DPAs [138], indicating that these channels function as thermoreceptors in these cells.

The findings described above provide ample evidence that external stimuli can activate the TRP channels expressed in odontoblasts. The odontoblasts may in turn activate the sensory nerves of the dental pulp. Recent studies have begun to provide insight into the mechanisms by which pulpal nerves are activated by stimulated odontoblasts. Activation of TRP channels and other receptors on the odontoblasts by external stimuli increases the intra-odontoblastic Ca^2+^ concentration [27,145,146,147,148]. These odontoblasts may then release ATP [27,140,151] and glutamate, which act on their receptors on adjacent nerve fibers of DPAs (Figure 2). ATP plays an important role in pain signaling through activation of purinergic receptors expressed on peripheral sensory nerve fibers [221,222]. Indeed, in an odontoblast/TG neuron co-culture preparation, mechanical stimulation of odontoblast-like cells increases the intracellular Ca^2+^ concentration in these cells as well as in the neighboring neuron-like cells [27]. Application of an inhibitor of ATP-permeable pannexin-1 (PANX-1) channels and ATP-degrading enzyme abolished the increases in intracellular Ca^2+^ in the neuron-like cells, but not in the mechanically stimulated odontoblast-like cells [27]. This suggests that ATP released through PANX-1 in stimulated odontoblasts excites neurons [27]. PANX-1 immunoreactivity in the cell bodies and the processes of odontoblasts has been observed [27]. In addition, purinergic receptor (P2X3/P2Y1/P2Y12) antagonists attenuate the increase in intracellular Ca^2+^ in neurons, but not in the mechanically-stimulated odontoblasts, suggesting that neuronal purinergic receptors are activated by the ATP released by the odontoblasts and its metabolite ADP [27]. Using the same odontoblast/TG neuron co-culture preparation, Sato et al. showed that mechanical stimulation of odontoblast-like cells evokes inward currents in medium-sized neuron-like cells that express NF-200 immunoreactivity (a marker of myelinated neurons), but not IB4 [223]. Medium-sized TG neurons (A-δ) have been implicated in sharp pain associated with dentine hypersensitivity [20,34,38]. Sato et al. also observed that the mechanical stimulation-induced currents were attenuated by application of a cocktail of TRPV1, TRPV2, TRPV4 and TRPA1 channel antagonists, suggesting involvement of these channels in transduction of mechanical stimuli applied to the odontoblast-like cells [223]. Furthermore, application of a P2X3 antagonist attenuated the induced currents in the neuron-like cells, suggesting activation of P2X3 on the neurons by ATP released from mechanically-stimulated odontoblasts [223]. PANX channels have also been detected in the dental pulp [224]. Expression of PANX-1 [27,224] and 2 [224] was observed in odontoblasts and their processes. ATP is hydrolyzed to ADP and other metabolites by ectonucleoside triphosphate diphosphohydrolases (NTPDases) [225]. NTPDase expression has been detected in the odontoblasts and Schwann cells that surround the myelinated pulpal nerves [226]. Functional NTPDase enzymatic activity is observed in odontoblasts and their processes, the sub-odontoblast layer, blood vessels and Schwann cells that surround the myelinated pulpal nerves, suggesting that ATP and its metabolites are present in the dental pulp [224]. Using an in vitro human tooth perfusion model, it has been demonstrated that mechanical or cold stimulation of the exposed dentine releases ATP from the dentine pulp complex, and that application of a PANX channel blocker reduces ATP release [224]. ATP can activate the ionotropic purinergic receptor P2X [227]. A variety of P2X receptor subtypes have been detected in the neurons of the TG [228,229]. Among these, P2X3 expression is comparatively high [229], and it has been detected in the nerves of the dental pulp [229,230,231,232]. ADP can activate the metabotropic purinergic receptor P2Y [227]. Expression of P2Y12 receptors is detected in the neurons of the TG [233].

Glutamate may also function as a signaling molecule in odontoblast–TG neuron communication [234]. In odontoblast/TG neuron co-culture preparations, the odontoblast mechanical stimulation-induced increase in Ca^2+^ in the neighboring neuron-like cells is attenuated by the application of a cocktail of antagonists of metabotropic glutamate receptors (mGluRs) [234]. When an ATP-degrading enzyme was incorporated into this cocktail, the neuronal Ca^2+^ increases were further suppressed, suggesting that both ATP and glutamate released by the odontoblasts act in a paracrine manner to signal neighboring neurons [234]. The Ca^2+^ increase in the neighboring neurons, but not in the stimulated odontoblasts, was also reduced by application of antagonists of glutamate-permeable anion channels, suggesting that glutamate may be released from the stimulated odontoblasts through these channels [234]. Furthermore, in the same study, odontoblast-like cells were observed to express group I, II and III mGluRs [234]. Glutamate is detected in the odontoblasts and nerve fibers of the rat dental pulp [235]. The nerve fibers also express mGluR5, which is upregulated following dentine injury [235]. The expression of mGluR5 has also been reported on TRPV1-immunoreactive nerve fibers in the human dental pulp [139]. These observations suggest that glutamate released from stimulated odontoblasts signal through mGluRs expressed on nearby nerve fibers (Figure 2).

**Figure 3:**
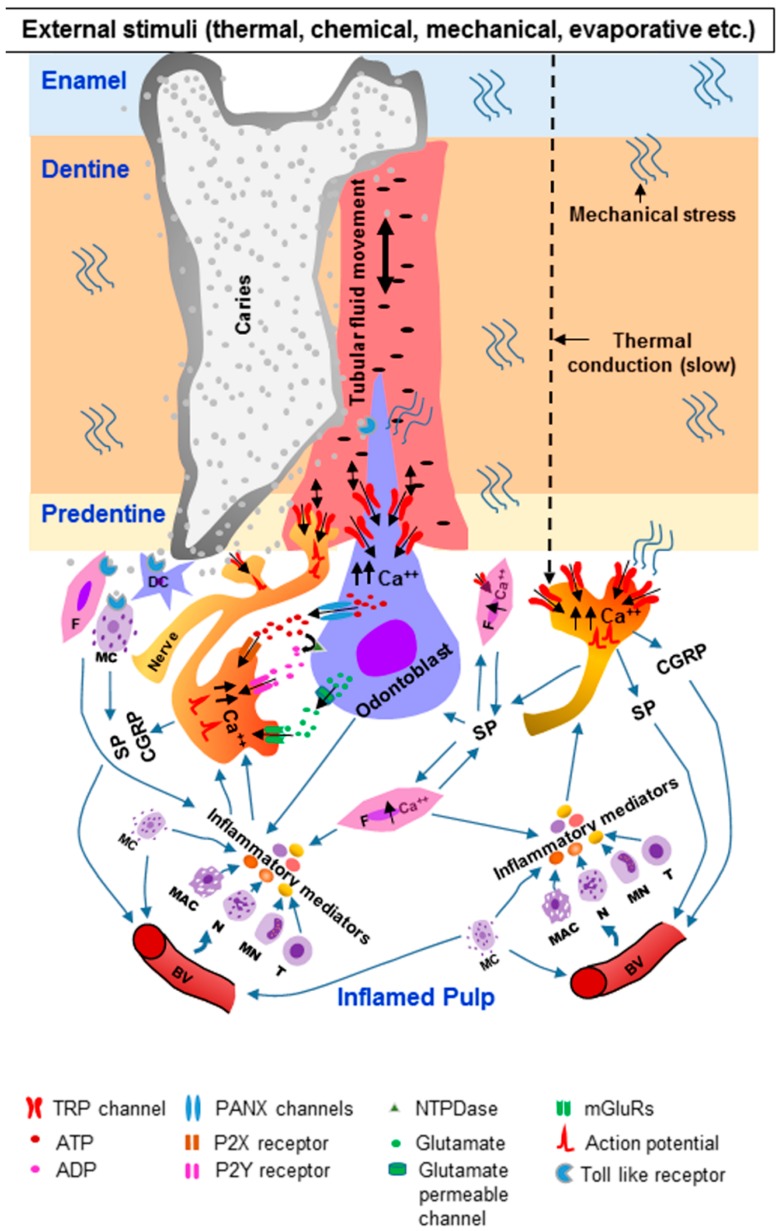
Involvement of TRP channels in the transduction of dental pain under inflammatory conditions. In caries-induced pulpitis, the various structures of the dentine–pulp complex (e.g., odontoblasts, fibroblasts, dendritic cells and resident mast cells etc.) sense the invading pathogens through specialized pattern recognition receptors, such as toll-like receptors (TLRs), leading to the initiation of an immune response. Blood-borne immune cells (e.g., neutrophils, monocytes and T-lymphocytes) infiltrate (indicated by a blue arrow) the pulp from the dilated blood vessels. These immune cells as well as odontoblasts and fibroblasts release (indicated by blue arrows) various inflammatory mediators that activate (indicated by blue arrows) cognate receptors on the nerve fibers, leading to sensitization. Sensitization can involve numerous changes, including enhancement of TRP sensitivity to external stimuli and increased expression on the nerve terminals by mechanisms such as post-translational modification and altered trafficking of these channels. Upregulation of TRP channels is observed in the odontoblasts and the pulpal nerves. The sensitized pulpal nerves release (indicated by blue arrows) various neuropeptides, such as substance P and CGRP. Neuropeptides can also be released (indicated by blue arrows) from fibroblasts and various immune cells. Local elevation of neuropeptides increases the release of inflammatory mediators from blood vessels that further elevate the release of neuropeptides from activated nerve fibers, exacerbating neurogenic inflammation. Besides, bacterial endotoxins can directly activate TRP channels on DPAs or odontoblasts and thereby contributed to the development of pain. SP: substance P; CGRP: calcitonin gene-related peptide; F: fibroblast; DC: dendritic cell; MC: mast cell; MAC: macrophage; T: T-lymphocyte; N: neutrophil; BV: blood vessel.

TRP channels in the DPAs may play an important role in pain transduction under inflammatory conditions of the dental pulp (pulpitis) (Figure 3).

In symptomatic pulpitis, the tooth is hypersensitive to external stimuli and pain persists after removal of the stimuli (lingering pain). The tooth can be spontaneously painful [6,7]. The pain may be caused by the sensitization of DPAs. Upregulation of various channels, including TRPs, in the odontoblasts and DPAs may lead to hyperexcitability of the nerves. Indeed, TRPV1 in the DPAs is up-regulated by LPS, a product of Gram-negative bacteria [135]. Upregulation of TRPV1 [141] and TRPA1 [149,196] in the nerve fibers of the dental pulp is observed in carious human teeth. In addition, upregulation of TRPA1 [195] and TRPV4 [142] in the pulpal nerve fibers is observed in teeth with signs of pulpitis. TRPA1 expression is also increased in the TG following experimental exposure of the dental pulp [194]. In caries-induced pulpitis, the various structures of the dentine–pulp complex (e.g., odontoblasts, fibroblasts, dendritic cells, resident mast cells, endothelial cells in blood vessels, nerve fibers) sense the invading pathogen-associated molecular patterns shared by microorganisms through specialized pattern recognition receptors, such as toll-like receptors (TLRs) and nucleotide-oligomerization binding domain (NOD)-like receptors [236,237,238,239], leading to the initiation of an immune response. Vasodilation and extravasation ensue, leading to the infiltration of blood-borne immune cells, such as neutrophils, monocytes and T-lymphocytes, into the pulp [240,241]. Various inflammatory and immune mediators (e.g., prostaglandins, bradykinin, histamine, cytokines, chemokines) are released from these cells (Figure 3). Activated odontoblasts, fibroblasts and mast cells also release inflammatory mediators [240,241]. These inflammatory mediators act on their receptors expressed on the nerve fibers, leading to sensitization of peripheral nerves [32,33,240,241,242,243]. The inflammatory mediators can also modulate the sensitivity of TRP channels to external stimuli [88,244,245,246]. They signal through their receptors on the sensory nerves, leading to the activation of intra-neuronal signaling pathways (e.g., protein kinases A and C, Src kinase, phospholipase C (PLC), extracellular signal-regulated kinase (ERK)) that induce post-translational modifications on multiple TRP channel proteins, thereby affecting their trafficking to the membrane, channel gating and sensitivity to various stimuli [88,244,246]. Additionally, several growth factors produced during inflammation (e.g., NGF) increase the production and transport of TRP channels to peripheral nerve terminals [88,244,246]. Growth factors may also directly increase the sensitivity of the nerves to stimuli [88,244,246]. The sensitized nerves in turn release various neuropeptides, such as substance P, CGRP and vasoactive intestinal peptide [240,241,242,243,246]. Neuropeptides, such as substance P, are also released from fibroblasts [247,248,249,250]. Expression of the mRNAs for substance P and its receptor neurokinin-1 has been reported in pulpal fibroblasts, suggesting that substance P may be released from and signal in an autocrine manner in these cells [247]. Substance P can also be released from various immune cells [251]. Local elevation of CGRP and substance P enhances vasodilation and immune cell invasion, further increasing the release of inflammatory mediators, thereby perpetuating and exacerbating the neurogenic inflammation (Figure 3) [32,242,247,248,249,250]. Under this inflammatory state, the threshold for activation of TRP channels may decrease, causing hypersensitivity of the tooth to external stimuli.

Interestingly, bacterial components can directly activate neurons before bacterial-induced immune response matured [252,253,254]. Gram-positive bacterial-derived factors (e.g., N-formylated peptides and α-hemolysin toxin) reported to elicit Ca^2+^ influx directly in mouse nociceptive neurons and contributed to the development of hypersensitivity in vivo [253]. Gram-negative bacterial toxin, lipopolysaccharide (LPS), also reported activating cultured TG neurons and sensitize TRPV1 channels via a toll-like receptor 4 (TLR4) mediated mechanism [254,255,256]. In a rat model, TLR4 signaling in the TRPV1 expressing TG neurons was implicated for acute dental pulpitis induced pain [257]. Recent studies demonstrate that LPS can activate TRP channels in a TLR4-independent manner [254,258]. It observed in a study that LPS directly activates TRPA1 channels present in the sensory neurons of the nodose and TG in a TLR4-independent mechanism [258]. Pain and neurovascular responses to LPS, including neuropeptide (CGRP) release were dependent on TRPA1 channel activation in the sensory neurons [258]. Another study demonstrates that in addition to TRPA1, LPS can directly activate other TRP channels (TRPV1, TRPM3, TRPM8) present in the sensory neurons [259]. These findings suggest that bacterial products can directly activate sensory nerve fibers before the development of neurogenic inflammation secondary to the immune response to infection [254]. Similarly, in caries-induced pulpitis, bacterial products may directly activate pulpal nerve fibers and contribute to the development of pain (Figure 3). LPS was also observed to activate TRPV4 channels on nonneuronal airway epithelial cells and increased intracellular Ca^2+^ concentration [260]. Since TRPV4 channels are expressed in the odontoblasts, there is a possibility of activation of TRPV4 on the odontoblasts by the bacterial toxin, which increases the intra-odontoblastic Ca^2+^ concentration and in turn, activates the intra-dental sensory neurons.

During inflammation, pulpal nerves undergo sprouting, which may also contribute to tooth hypersensitivity [38,261]. NGF may stimulate nearby pulpal nerves to sprout new branches [38,261], which may in turn increase the number of TRP channels in the dental pulp. Sprouting of the nerves may also lead to innervation of more dentinal tubules, further increasing pain sensitivity [38,261].

Under inflammatory conditions, extravasation of fluid from blood vessels can elevate the pressure within the pulp, because dental pulp is enclosed by hard tissues, creating a low compliance environment [261]. It has been reported that pulpal tissue pressure during inflammation can rise from 15 to 40–50 cm H2O [261,262]. This increase in intra-pulpal pressure can excite the mechanoreceptors (including mechanosensitive TRP channels) on the nerve fibers of the pulp and lead to spontaneous pain.

Fibroblasts in the dental pulp are also reported to be involved in pulpitis. Fibroblasts are abundantly present in the dental pulp and are responsible for the synthesis of extracellular matrix and the maintenance of the structural integrity of the dental pulp [6,263,264]. They are also reported to produce pro-inflammatory cytokines, including interleukin (IL)-1β, IL-6 and IL-8, in response to bacterial stimulation [265,266]. Pro-inflammatory cytokines can be released from fibroblasts by neuropeptides [267,268,269]. Pulpal fibroblasts also express TRPV1, and activation of this channel leads to the release of IL6, suggesting a role in the development of pulpitis [209]. TRPM2 expression is also increased in the pulpal fibroblasts of teeth with signs of irreversible pulpitis [211].

## 5. Clinical Significance

Despite the high prevalence of dental pain, effective pain management is lacking because the cellular and molecular mechanisms underlying the pain are unclear, particularly those involved in the transduction of nociceptive signals. Elucidation of these transduction mechanisms is crucial for the development of therapeutic strategies that target the root cause of dental pain. Pharmacologically targeting TRP channels is a novel therapeutic strategy for managing dental pain. Various studies show that pharmacological antagonists of TRP channels attenuate the experimental stimuli-induced increases in intracellular Ca^2+^ concentration in odontoblasts and DPAs. TRP channels in the sensory neurons have been targeted to develop pain-specific local anesthesia in an animal study [270]. Capsaicin (a TRPV1 activator) has been combined with a permanently charged derivative of lidocaine, QX-314, and this combination appears to be effective as a local anesthetic [271,272,273]. An animal study demonstrated that local application of this drug combination in the gingiva near a tooth before extraction reduced extraction-induced neuronal activation, indicated by reduced expression of c-Fos in the brainstem trigeminal subnucleus caudalis [273]. Eugenol was reported to sensitize and then desensitize TRPV1 channels, which may explain the pain suppressing action of zinc oxide eugenol cements used in temporary restorations of carious teeth [274]. Several small-molecule antagonists of TRPV1 channels have been tested in human clinical trials for dental pain, but unfortunately, outcomes have been poor or the clinical trials were prematurely terminated [275,276,277] due to side-effects. In one clinical trial, the analgesic effect of the first-generation TRPV1 antagonist AMG 517 (Amgen) was tested following extraction of a third molar tooth. However, the drug increased core body temperature, resulting in termination of the trial [276]. Another TRPV1 antagonist (AZD1386, Astra-Zeneca) was tested in patients with acute pain following removal of the mandibular third molar, but displayed only a short-term analgesic effect [277]. Further studies are required to identify more effective analgesics targeting TRP channels. Incorporation of pharmacological antagonists of TRP channels to dentine desensitizing formulations, dental cements and pulp capping materials is a potential therapeutic strategy for dental pain.

## 6. Conclusions

The sensory detection system in the tooth is unique. Any type of external stimuli on the exposed dentine or tooth with pulpitis predominantly causes pain sensation. However, the underlying pain transduction mechanisms are not yet fully understood. Various TRP channels that have been detected in the odontoblasts and DPAs may play an important role in the transduction of external stimuli to electrical signals in the nerves, which are conveyed to and perceived as pain by the brain. The TRP channels may serve as potential drug targets for the development of pharmacological strategies to manage dental pain.
